# An eye-tracking-based Stroop test: an efficient method for evaluating frontal lobe function

**DOI:** 10.3389/fnagi.2026.1787430

**Published:** 2026-05-04

**Authors:** Sho Yamamoto, Shuko Takeda, Sho Miki, Shin Teshirogi, Yoshinobu Kishino, Mizuki Katsuhisa, Momoko Okawara, Akane Oyama, Yuki Ito, Tsuneo Nakajima, Yoichi Takami, Yasushi Takeya, Koichi Yamamoto, Ryuichi Morishita

**Affiliations:** 1 Department of Geriatric and General Medicine, Graduate School of Medicine, Osaka University, Osaka, Japan; 2 Osaka Psychiatric Research Center, Osaka Psychiatric Medical Center, Osaka, Japan; 3 Department of Clinical Gene Therapy, Graduate School of Medicine, Osaka University, Osaka, Japan; 4 Division of Health Sciences, Graduate School of Medicine, Osaka University, Osaka, Japan

**Keywords:** attention, dementia, eye-tracking technology, frontal lobe function, screening, Stroop effect

## Abstract

**Introduction:**

Assessing attention and frontal lobe function is essential for diagnosing dementia. The Stroop test is widely used for these purposes, and its validity and reliability have been verified. Nevertheless, test-related burdens and procedural complexity have prevented their use as a screening tool. We developed a novel Stroop test using eye-tracking technology and evaluated its validity and utility in a clinical setting.

**Methods:**

The study included 97 older adults at a memory clinic. Participants completed both the eye-tracking-based and conventional paper-based Stroop tests, along with MMSE, FAB, and TMT-A/B.

**Results:**

Scores on the eye-tracking-based Stroop test significantly correlated with the paper-based test (*r* = 0.634), FAB (*r* = 0.480), and TMT-B (*r* = −0.536). It achieved an AUC–ROC of 0.701 for frontal lobe dysfunction and 0.708 for dementia.

**Discussion:**

The eye-tracking-based Stroop test has the potential to efficiently assess frontal lobe function and serve as a screening tool for dementia.

## 1 Introduction

With the aging global population, the number of individuals with dementia is increasing and is projected to reach 153 million by 2050 (Nichols et al., 2022). As cognitive impairment becomes more severe, the associated costs rise accordingly (Tay et al., 2024), highlighting the importance of early intervention. However, many patients remain undiagnosed, due to the lack of efficient screening tools for detecting cognitive decline and limitations in healthcare access (Amjad et al., 2018). The development of low-cost, easily accessible screening tools is essential for enabling early diagnosis (Ge et al., 2021). As a result, growing attention has been directed toward non-invasive, rapid, and repeatable digital biomarkers to detect early signs of dementia.

Frontal lobe dysfunction, particularly attention deficits, is one of the early manifestations of dementia, along with memory impairment (Arvanitakis et al., 2019; McGuinness et al., 2010). Attention serves as the foundation for a wide range of cognitive functions and is considered to have a crucial impact on overall cognition (Dams-O'Connor and Gordon, 2013; Obonsawin et al., 2002). In particular, attention is closely related to executive function (Perry and Hodges, 1999). Frontal lobe dysfunction has been reported to predict the conversion from mild cognitive impairment (MCI) to dementia (Jung et al., 2020). These findings indicate that assessing frontal lobe function is particularly important in the evaluation of cognitive function.

Various neuropsychological tests have been used to assess frontal lobe function. Specifically, the utility of the Stroop test (Stroop, 1935), the Frontal Assessment Battery (FAB) (Dubois et al., 2000), and the Trail Making Test (TMT) (Bowie and Harvey, 2006) have been reported for this purpose, and multiple tests are often combined to increase sensitivity in clinical practice. Patients with frontal lobe dysfunction caused by a traumatic brain injury, cerebral infarction, and frontal lobe atrophy due to frontotemporal dementia are reported to have low scores on these tests (Johns et al., 2009; Kopp et al., 2015; Stuss et al., 2001; Valverde et al., 2009), supporting their validity as measures of frontal lobe function.

The Stroop test, first reported by Stroop (1935), is a neuropsychological assessment of frontal lobe function. It evaluates attention and executive function by measuring the interference between two conflicting pieces of information. Many versions, including the original, employ incongruence between the word that describes a color and the ink in which it is written (color-based Stroop test). In addition to the color-based Stroop test, other variants have been reported, including tasks based on location (Wühr, 2007), emotion (Frings et al., 2010), and numerical values (Henik and Tzelgov, 1982). The Stroop test is widely used in both clinical and research settings to assess a range of psychiatric and neurodegenerative disorders, including mood disorders such as depression (Epp et al., 2012), schizophrenia (Westerhausen et al., 2011), attention-deficit/hyperactivity disorder (Schreiber et al., 2014), Alzheimer’s disease (Ben-David et al., 2013), Parkinson’s disease (Sisco et al., 2016), brain injury (Ben-David et al., 2011), and hepatic encephalopathy (Bajaj et al., 2013).

To date, the Stroop test has been administered using various methods, most commonly in written or oral response formats (Macleod, 1991). More recently, Stroop tasks implemented on touchscreen devices (Roque et al., 2023) and personal computers (Pilli et al., 2013) have been reported, aiming to enhance the objectivity of input data and improve the accuracy of data processing. In addition, multiple scoring approaches have been proposed. For example, some methods measure the total time required to complete the task, whereas others use the number of correct responses within a fixed time limit or require participants to complete a predetermined number of trials. However, these approaches share several limitations, including the need for examiner expertise to administer the test and interpret the results, the existence of multiple scoring standards, and the substantial psychological burden placed on participants due to the large number of required responses.

We previously developed a novel cognitive assessment tool based on an eye-tracking system as a screening tool for global cognitive function (Katsuhisa et al., 2024; Oyama et al., 2019; Takami et al., 2025). In our eye-tracking-based cognitive assessment (ETCA), participants watch task movies consisting of instructions and multiple options to select. They find the correct option and respond by focusing on it. The cognitive score is calculated based on the percentage of viewing time spent on the correct option. The ETCA enables semi-automated administration and scoring, eliminating the need for examiner expertise and reducing inter-examiner variability—limitations inherent in traditional paper-based methods. Furthermore, because the test is not interview-based, participants can complete the assessment with low psychological stress, as they do not have to be concerned with making errors in their verbal responses. Eye-tracking technology has become increasingly accessible across various devices, suggesting that such assessments may be even more widely available in the coming years.

In this study, we developed an eye-tracking-based Stroop test, termed iStroop, and evaluated its clinical utility as a rapid and objective screening tool for detecting frontal lobe dysfunction, in a cohort primarily consisting of older adults attending a memory clinic. First, we examined the correlations of scores between the eye-tracking-based Stroop test and a conventional paper-based Stroop test. Second, we evaluated the correlations of the scores between the eye-tracking-based Stroop test and well-validated and reliable neuropsychological tests for frontal lobe function (FAB and TMT). Third, we evaluated its diagnostic performance to detect frontal lobe dysfunction. Finally, we evaluated its utility as a screening tool for detecting MCI and dementia.

## 2 Materials and methods

### 2.1 Participants

The study cohort included 97 participants recruited at Osaka University Hospital. All participants underwent standard physical and neurological examinations, neuropsychological assessments [The Mini-Mental State Examination (MMSE), FAB, TMT-A/B, paper-based Stroop test (Watanabe et al., 2011)] and the eye-tracking-based Stroop test. All neuropsychological tests and the eye-tracking-based Stroop test were performed within the same week. Participants with MCI and dementia underwent standard blood tests to rule out other disorders that may cause cognitive impairment, such as thyroid disorder or vitamin deficiencies. Participants were classified into three groups based on their MMSE and Clinical Dementia Rating (CDR) scores. The criteria for MMSE-based classification are described in Section 2.7 (Neuropsychological tests). For the CDR-based classification, participants were categorized as cognitively normal (CN) with a CDR of 0, MCI with a CDR of 0.5, and dementia with a CDR of 1 or higher. Participants with active psychiatric disorders or severe visual impairments were excluded from the study. We confirmed verbally that participants had no history of color blindness.

All participants and their families provided written informed consent. All study protocols were approved by Osaka University Hospital’s Institutional Review Board, and ethical approval was provided by the Ethics Committee at Osaka University. All research was performed in accordance with relevant guidelines and regulations, including the Declaration of Helsinki.

### 2.2 Experimental protocol

The assessments were conducted by one examiner and the participant in a quiet neuropsychological testing room with normal illumination. The room was equipped with a standard desk for neuropsychological testing and an eye-tracking device. The conventional paper-based Stroop test and the eye-tracking-based Stroop test were administered on the same day in this specific order. After completing the paper-based Stroop test, participants were given a rest period of a few minutes before proceeding to the eye-tracking-based Stroop test in the same room. For the eye-tracking-based Stroop test, participants were instructed simply to look at the task movies on the monitor; no verbal or pointing responses were required. They were also asked to remain as still as possible during the approximately 3-min testing period, although strict head fixation was not required. Following the completion of the assessments, data analysis was performed by researchers who were independent of the test administration. Further details regarding the specific procedures for both the paper-based and eye-tracking-based Stroop tests are provided in the subsequent sections.

### 2.3 Eye-tracking device

A high-performance eye tracker utilizing the corneal reflection method (Gazefinder NP-100; JVC KENWOOD Corporation, Japan) was used to record gaze data, as described previously (Katsuhisa et al., 2024; Oyama et al., 2019). Briefly, the device uses infrared light sources and cameras located beneath a 19-inch monitor to detect the participant’s gaze points at a frequency of 50 Hz.

### 2.4 Preparation for the assessment

The participant was seated in front of a monitor and instructed to watch the task movies displayed on it. After a brief calibration for gaze detection, a series of task movies was presented on the monitor, as previously described (Katsuhisa et al., 2024; Oyama et al., 2019).

### 2.5 The task movies for the eye-tracking-based Stroop test

**Figure 1:**
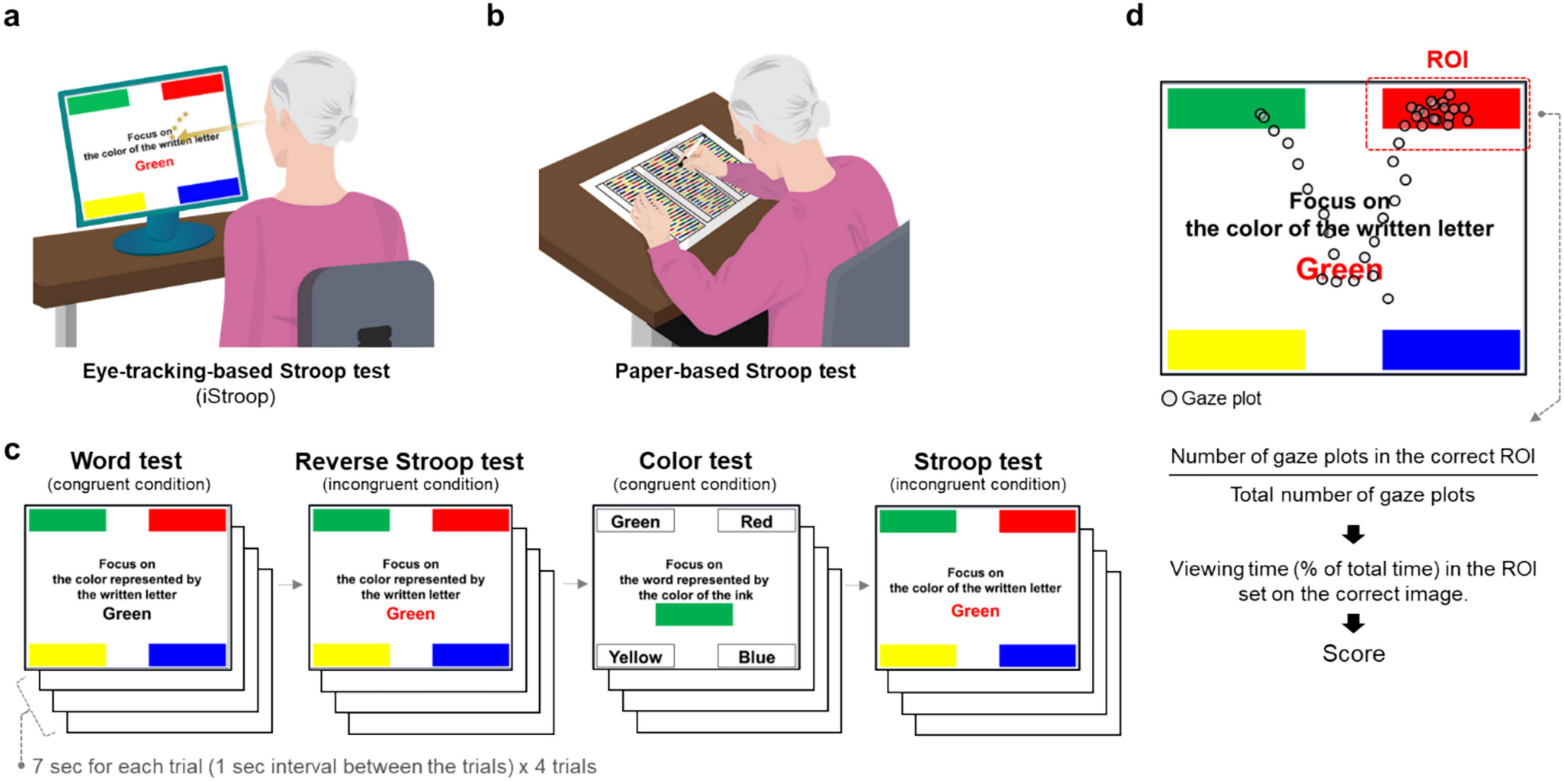
Overview of the eye-tracking-based Stroop test. Schematic representations of the **(a)** eye-tracking-based and **(b)** traditional paper-based Stroop test. **(c)** Task movies for the eye-tracking-based Stroop test consisted of 16 trials from four distinct conditions (word test, Reverse Stroop test, color test, and Stroop test): each condition had four trials. **(d)** Scoring method. Participants’ gaze points were recorded as gaze plots and the number of them in the ROI of the correct option was calculated. The percentage of viewing time spent on the correct option was used as the score for each trial. ROI, region of interest.

Task movies for the eye-tracking-based Stroop test consisted of 16 trials from four distinct conditions (word test, Reverse Stroop test, color test, and Stroop test): each condition had four trials with no repetition for any color (Figure 1). The different types of stimuli were presented in a blocked order (all trials of a specific condition in a sequence). Each trial was presented for 7 s with 1-s interval between trials. The instruction “Next, a different type of task will be presented” was presented for 2 s between blocks (i.e., when moving to the next condition).

### 2.6 Data processing for scoring

A region of interest (ROI) was set on the correct option for each trial. Instead of relying on a conventional dichotomous outcome (i.e., correct or incorrect) based on a final discrete selection, the raw percentage of viewing time spent in the correct ROI within the 7-s timeframe was utilized as a continuous accuracy score (Figure 1). Using this continuous metric allows for the evaluation of intermediate cognitive states, such as the degree of uncertainty during the cognitive interference process, thereby providing greater information than a simple final selection. The percentage of viewing time in the correct ROI from each trial was averaged and used as the total score for the eye-tracking-based Stroop test.

### 2.7 Neuropsychological tests

The MMSE was used to assess global cognitive function and was available for all 97 participants. MMSE scores range from 0 to 30, with lower scores indicating greater impairment. Based on MMSE score cutoffs as previously reported (Sugishita et al., 2010), participants were classified into three groups: MMSE-defined CN (scores of 28–30), MMSE-defined suspected MCI (scores of 24–27), and MMSE-defined suspected dementia (scores of 23 or lower). The FAB and TMT were used to examine frontal lobe functions (Bowie and Harvey, 2006; Dubois et al., 2000). FAB scores range from 0 to 18, with lower scores indicating greater impairment and were available for 94 participants. FAB scores were unavailable for three participants. The cutoff value for frontal lobe dysfunction (≤11/18) was used, as previously reported (Slachevsky et al., 2004). The TMT consists of two components, parts A and B; longer completion times indicate greater impairment. The results of the TMT were not available from 12 participants for the TMT-A and 26 participants for the TMT-B, due to test discontinuation according to participants’ requests or predefined termination criteria. The cutoff values for impaired frontal lobe function were used as previously reported (Tombaugh, 2004).

### 2.8 Paper-based Stroop test

Paper-based Stroop test comprised four conditions: Word test, Reverse Stroop test, Color test, Stroop test and administered as previously reported (Hakoda, 1990; Song and Hakoda, 2011). A series of items were printed on sheets of paper and clustered by condition. Participants were instructed by examiners to respond to each task by placing a check mark on the answer sheet with a pencil (Figure 1b). The score for each condition was determined by counting the number of correct responses in a given amount of time (40 s). Participants were given a 10-s practice session before starting each condition, which consisted of the same kind of stimuli as test session.

### 2.9 Statistical analysis

Differences in MMSE scores, FAB scores, and TMT completion times between the two groups were analyzed using the Mann–Whitney *U* test. Spearman’s rank correlation was applied to determine the correlation between the eye-tracking-based Stroop scores and the results of the neuropsychological tests (conventional paper-based Stroop test, FAB, TMT, MMSE). The diagnostic performances of the eye-tracking- and paper-based Stroop tests for detecting frontal lobe dysfunction and dementia were examined using an ROC analysis. An area under the ROC curve was used as an index of diagnostic performance. Data analysis was performed using Python (version 3.11.7) and scientific computing libraries (matplotlib, numpy, pandas, scipy, sklearn). Data were plotted using matplotlib in Python. *p* < 0.05 was considered significant.

## 3 Results

### 3.1 Overview of the eye-tracking-based Stroop test

Figure 1 shows an overview of the eye-tracking-based Stroop test. We compared the scores from eye-tracking- (Figure 1a) and traditional paper-based (Figure 1b) Stroop tests. Task movies for the eye-tracking-based Stroop test consisted of a total of 16 trials from four distinct conditions (word test, Reverse Stroop test, color test, and Stroop test). Each condition had four trials with no repetition for any color (Figure 1c). The task movies were presented sequentially on the screen. Each trial had instruction text in the center of the screen and four distinct options in each corner, comprising one correct answer and three incorrect answers (Figure 1d). Participants viewed the task movies and were instructed to find and focus on the correct option. We calculated viewing time (% of total time) spent in the ROI that was set on the correct option based on the gaze plots recorded during the assessment (Figure 1d). The percentages of the viewing time spent on the correct option during each trial were averaged and used as scores for the test.

### 3.2 Participant characteristics

**Figure 2:**
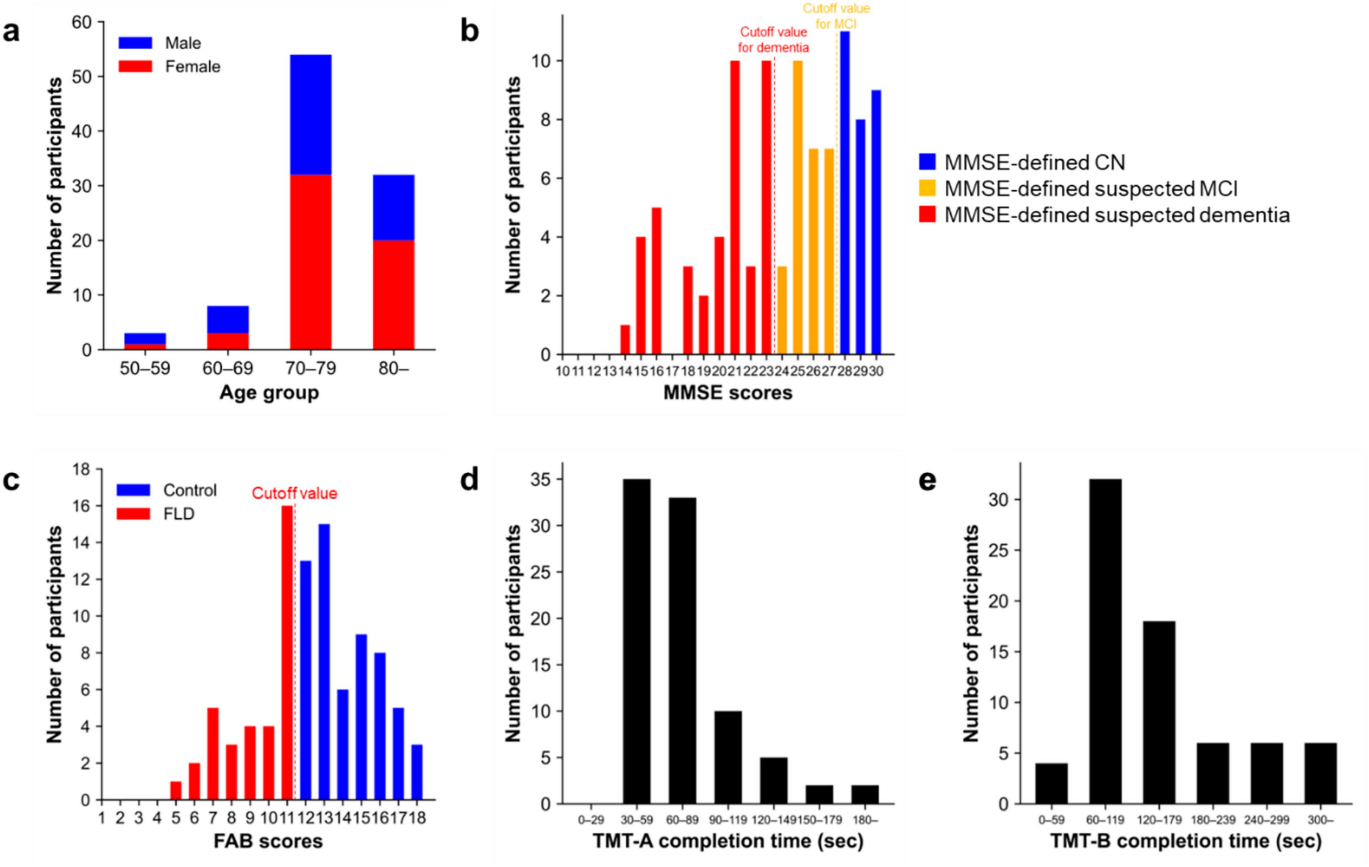
Neuropsychological test scores distribution. Distributions of participants’ **(a)** age, **(b)** MMSE scores, **(c)** FAB scores, **(d)** TMT-A completion times, and **(e)** TMT-B completion times. In **(b)**, the vertical dashed lines in the MMSE scores’ distribution represent the traditional cutoff values for detecting suspected MCI (yellow dashed line) and suspected dementia (red dashed line). Blue, yellow, and red bars indicate participants with scores higher than the cutoff values for MCI (MMSE-defined CN), those with suspected MCI, and those with suspected dementia, respectively. In **(c)**, the vertical dashed line in the FAB scores’ distribution represents the traditional cutoff value for detecting FLD. CN, cognitive normal; FAB, frontal assessment battery; FLD, frontal lobe dysfunction; CN, cognitively normal; MMSE, Mini-mental state examination; MCI, mild cognitive impairment; TMT, Trail Making Test.

The distributions of participants’ ages and neuropsychological test scores are presented in Figure 2. The study included a total of 97 participants (41 males, 56 females), with a mean age of 76.4 years (SD = 7.1) (Figure 2a). The MMSE scores ranged from 14 to 30 (mean = 24.1, SD = 4.4); 28% (27/97) were suspected of having MCI and 43% (42/97) were suspected of having dementia according to the traditional cutoff values (Figure 2b). The participant characteristics for the MMSE-defined CN, suspected MCI, and suspected dementia groups are presented in Supplementary Table 1. The FAB scores ranged from 5 to 18 (mean = 12.4, SD = 3.0); 37% (35/94) were suspected of having frontal lobe dysfunction according to the traditional cutoff value (Figure 2c). The mean completion times for the TMT-A and TMT-B were 77.3 (SD = 42.0) (Figure 2e) and 152.8 s (SD = 97.3) (Figure 2e), respectively. The participant characteristics for the CDR-defined CN, MCI, and dementia groups are presented in Supplementary Table 2.

### 3.3 Correlations in scores between the eye-tracking-based and conventional paper-based Stroop tests

**Figure 3:**
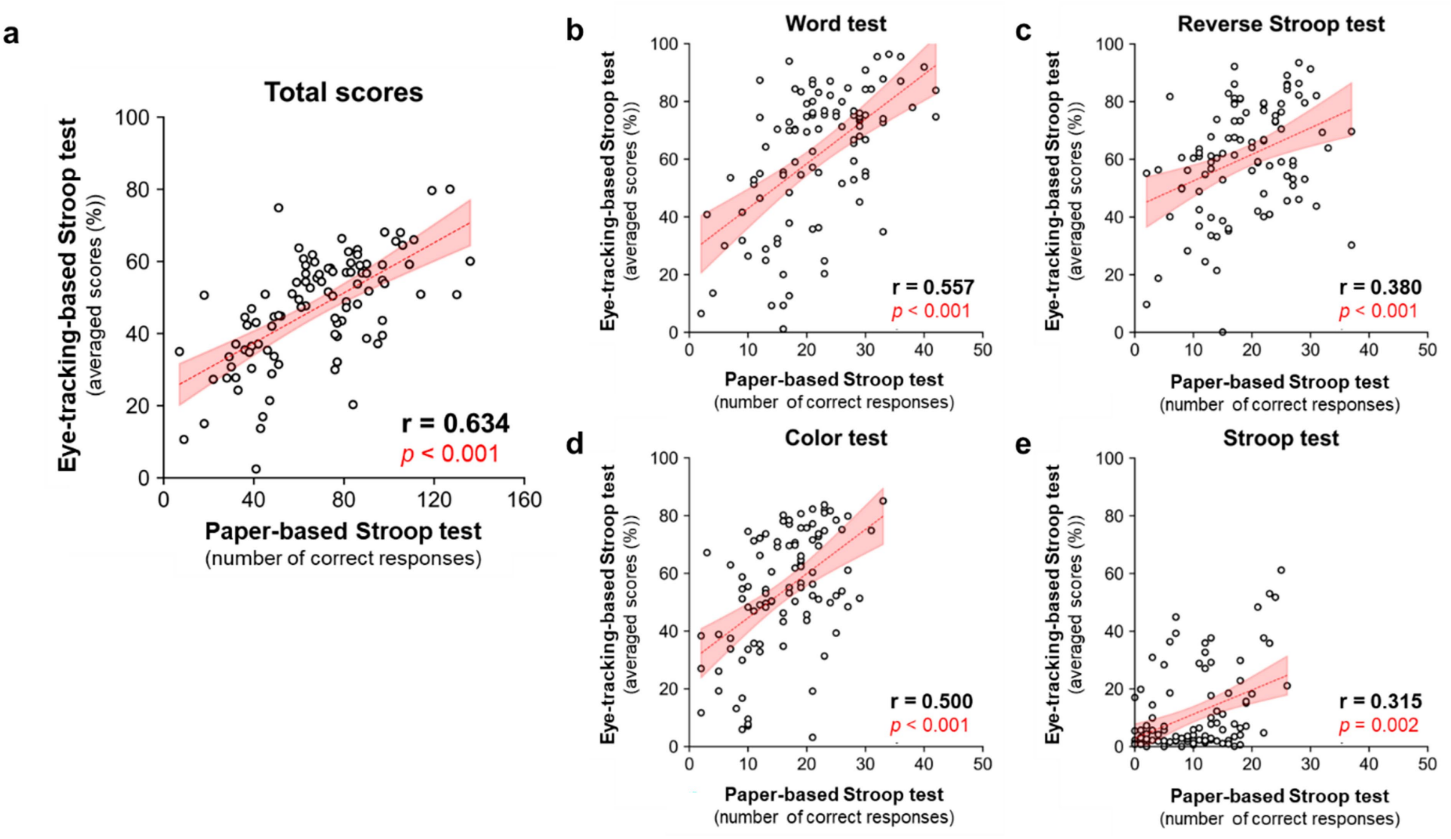
Correlations of scores between the eye-tracking- and conventional paper-based Stroop tests. **(a)** Scatter plot analysis for correlations in total scores between the eye-tracking- and conventional paper-based Stroop tests. *p* < 0.001, Spearman’s rank test, *n* = 97. **(b–e)** Scatter plot analyses for correlation in the scores of each condition [**(b)** word test, **(c)** Reverse Stroop test, **(d)** color test, and **(e)** Stroop test] between the eye-tracking- and conventional paper-based Stroop tests. A linear trendline is superimposed on the data, along with a 95% confidence interval of the trendline colored in shaded red shading. *p* < 0.001, Spearman’s rank test, *n* = 97.

We evaluated the correlations in scores between the eye-tracking- and conventional paper-based Stroop tests to assess their concurrent validity (Figure 3). The total scores of the eye-tracking-based Stroop test showed a significant positive correlation with the total scores of the conventional paper-based Stroop test (Figure 3a, *r* = 0.634, *p* < 0.001, Spearman’s rank correlation). The scores in each condition (word test, Reverse Stroop test, color test, and Stroop test) also showed significant positive correlations between the eye-tracking- and paper-based Stroop tests (Figure 3b [word test], *r* = 0.557; Figure 3c [Reverse Stroop test], *r* = 0.380; Figure 3d [color test], *r* = 0.500; Figure 3e [Stroop test], *r* = 0.315; *p* < 0.001, Spearman’s rank test). These results supported the equivalence of the eye-tracking-based Stroop test scores to conventional paper-based Stroop test scores.

### 3.4 Correlations in scores between the eye-tracking-based Stroop test and neuropsychological tests for frontal lobe function

**Figure 4:**
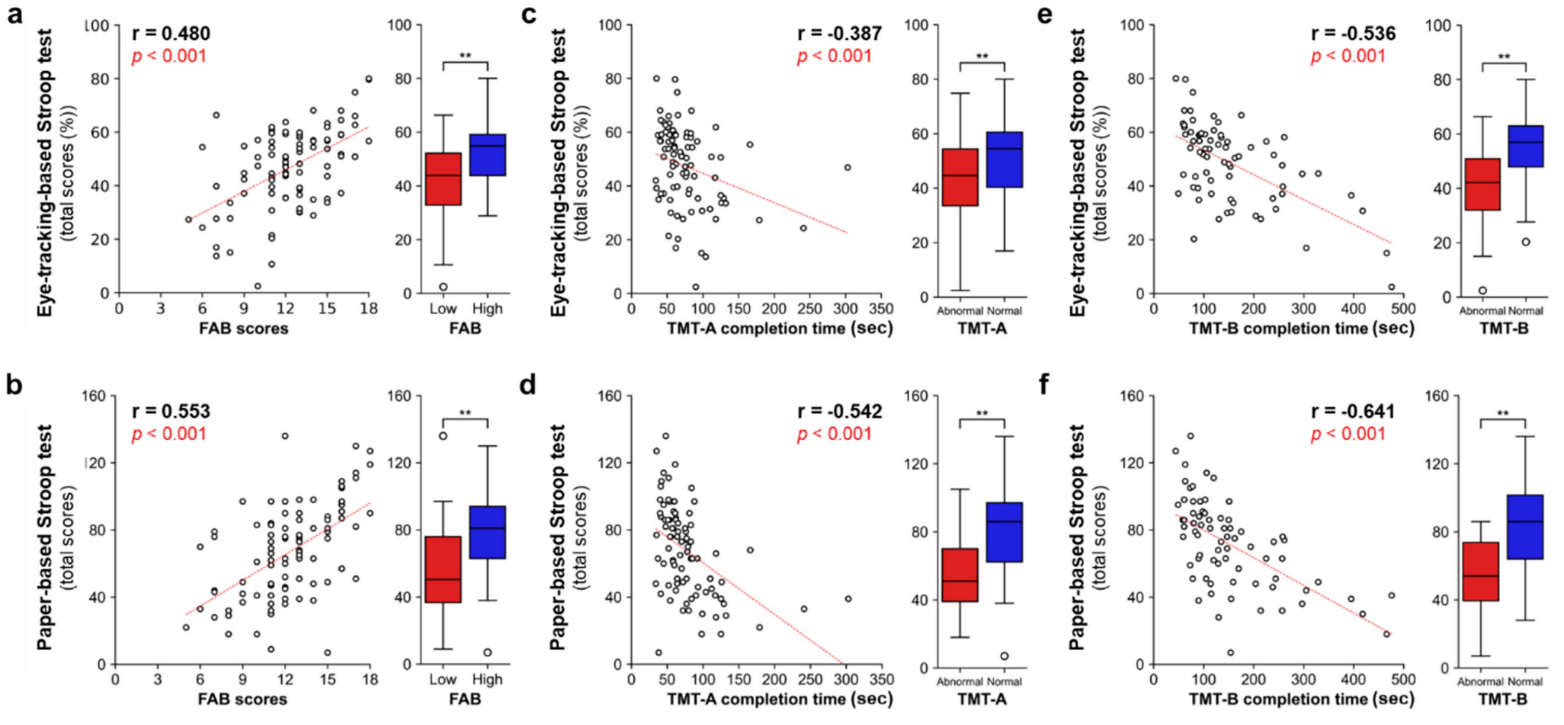
Correlations between the eye-tracking-based Stroop test and neuropsychological tests for frontal lobe function. **(a,b)** Scatter plot analyses for correlations in scores between the eye-tracking- (**a**, left panel) and paper-based (**b**, left panel) Stroop tests and FAB. *p* < 0.001, Spearman’s rank test, *n* = 94. Participants were divided into low (0–11, *n* = 35) and high (12–18, *n* = 59) FAB groups, based on the traditional cutoff value for frontal lobe dysfunction. Mann–Whitney *U* test. **p < 0.01. **(c,d)** Scatter plot analyses for correlations in scores between the eye-tracking- (**c**, left panel) and paper-based (**d**, left panel) Stroop tests and TMT-A. *p* < 0.001, Spearman’s rank test, *n* = 87. Participants were divided into abnormal (*n* = 45) and normal (*n* = 42) groups, based on the traditional cutoff value. Mann–Whitney *U* test. **p < 0.01. **(e,f)** Scatter plot analyses for correlations in scores between the eye-tracking- (**e**, left panel) and paper-based (**f**, left panel) Stroop tests and TMT-B. *p* < 0.001, Spearman’s rank test, *n* = 87. Participants were divided into abnormal (*n* = 30) and normal (*n* = 31) groups, based on the traditional cutoff value. Mann–Whitney *U* test. **p < 0.01. Box plots indicate the median (horizontal line), the interquartile range (25th–75th percentiles; box), and whiskers extending to 1.5× the interquartile range. Outliers are shown as individual points. FAB, Frontal Assessment Battery; TMT, Trail Making Test.

Next, we evaluated the correlations in scores between the eye-tracking-based Stroop test and well-validated neuropsychological tests as measures of frontal lobe function (FAB and TMT) to assess construct validity (Figure 4). Both the eye-tracking- (Figure 4a, left panel, *r* = 0.480) and paper-based (Figure 4b, left panel, *r* = 0.553) Stroop test scores showed significant positive correlations with FAB scores (*p* < 0.001, Spearman’s rank test, *n* = 97). Participants were divided into two groups based on the traditional cutoff value for frontal lobe dysfunction (low, 0–11/18; high, 12–18/18). Participants with low FAB scores showed significantly lower scores in both the eye-tracking- (Figure 4a, right panel) and paper-based (Figure 4b, right panel) Stroop tests (Mann–Whitney *U* test, *p* < 0.01). Both the eye-tracking- (Figure 4c, left panel, *r* = −0.387) and paper-based (Figure 4d, left panel, *r* = 0.542) Stroop test scores showed significant negative correlations with TMT-A completion times (*p* < 0.001, Spearman’s rank test, *n* = 87). Participants with abnormal TMT-A completion time showed significantly lower scores in both the eye-tracking- (Figure 4c, right panel) and paper-based (Figure 4d, right panel) Stroop tests (Mann–Whitney *U* test, *p* < 0.01). Both the eye-tracking- (Figure 4e, left panel, *r* = −0.536) and paper-based (Figure 4f, left panel, *r* = 0.641) Stroop test scores showed significant negative correlations with TMT-B completion times (*p* < 0.001, Spearman’s rank test, *n* = 87). Participants with abnormal TMT-B completion times showed significantly lower scores in both the eye-tracking- (Figure 4e, right panel) and paper-based (Figure 4f, right panel) Stroop tests (Mann–Whitney *U* test, *p* < 0.01).

### 3.5 Diagnostic performance of the eye-tracking-based Stroop test for frontal lobe dysfunction

**Figure 5:**
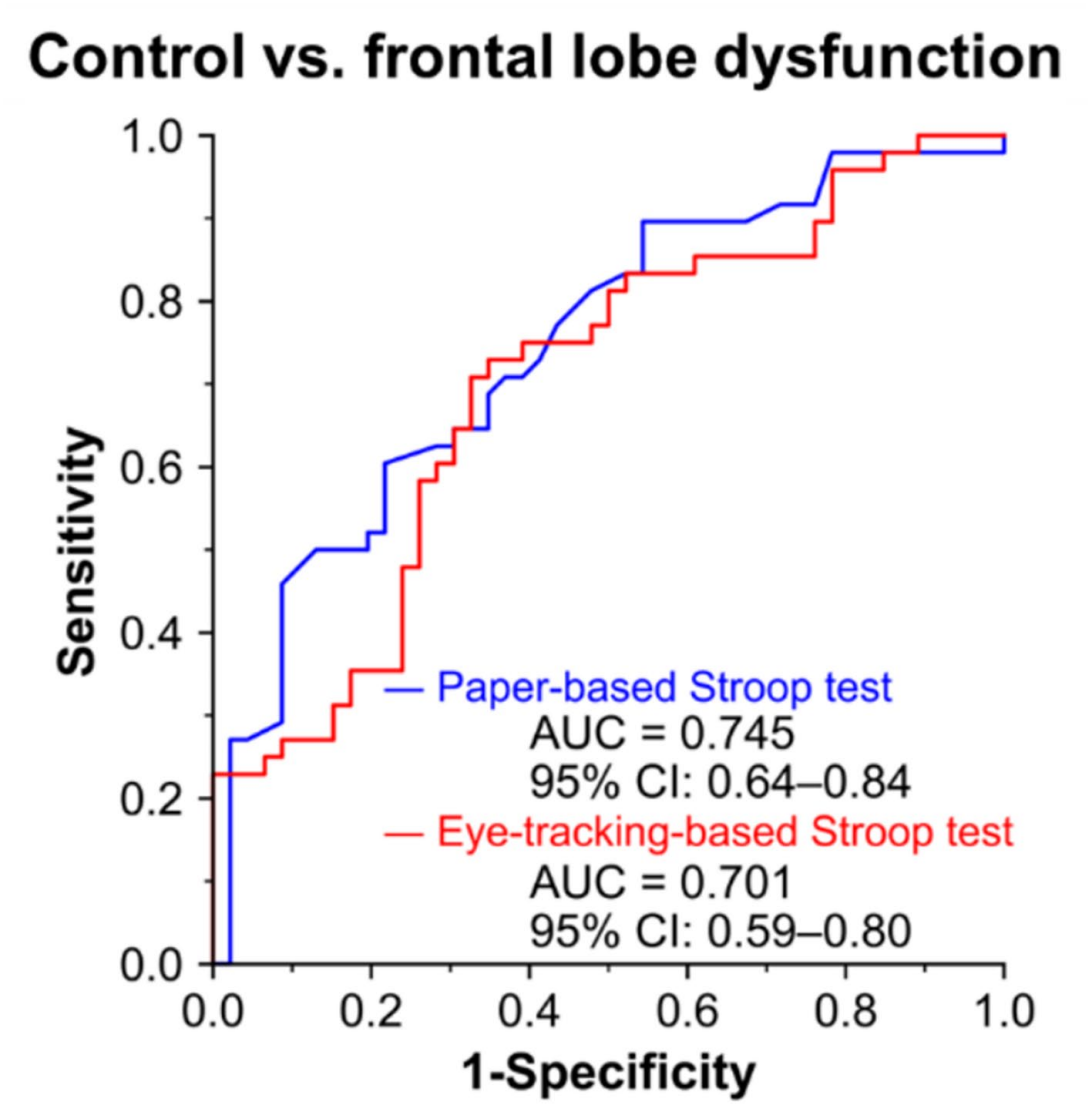
Diagnostic performances of the eye-tracking-based and paper-based Stroop tests for frontal lobe dysfunction. ROC curve analyses of diagnostic performances of the eye tracking-based Stroop test scores (red) and the conventional paper-based Stroop test scores (blue) for discriminating participants with frontal lobe dysfunction (FAB score ≤11, *n* = 35) from the control group (FAB score ≥ 12, *n* = 59). An area under the ROC curve (AUC) analysis was used to compare the diagnostic performances of the two tests. ROC, receiver operating characteristic.

We examined the diagnostic performance of the eye-tracking-based Stroop test for frontal lobe dysfunction (Figure 5). An ROC curve analysis was used to assess the performance of the eye tracking- and paper-based Stroop tests for diagnosing frontal lobe dysfunction. Frontal lobe dysfunction was defined based on the traditional cutoff value of an FAB score (≤11/18). 34 (37%) participants had FAB scores of 11 or lower and were thus regarded as having frontal lobe dysfunction. To discriminate participants with frontal lobe dysfunction from the control group, the eye-tracking-based Stroop test achieved an area under the curve (AUC) of 0.701 (95% CI 0.58–0.80), which was comparable with the conventional paper-based Stroop test (AUC = 0.745, 95% CI 0.64–0.84) (Figure 5).

### 3.6 Performance of the eye-tracking-based Stroop test in detecting global cognitive impairment

**Figure 6:**
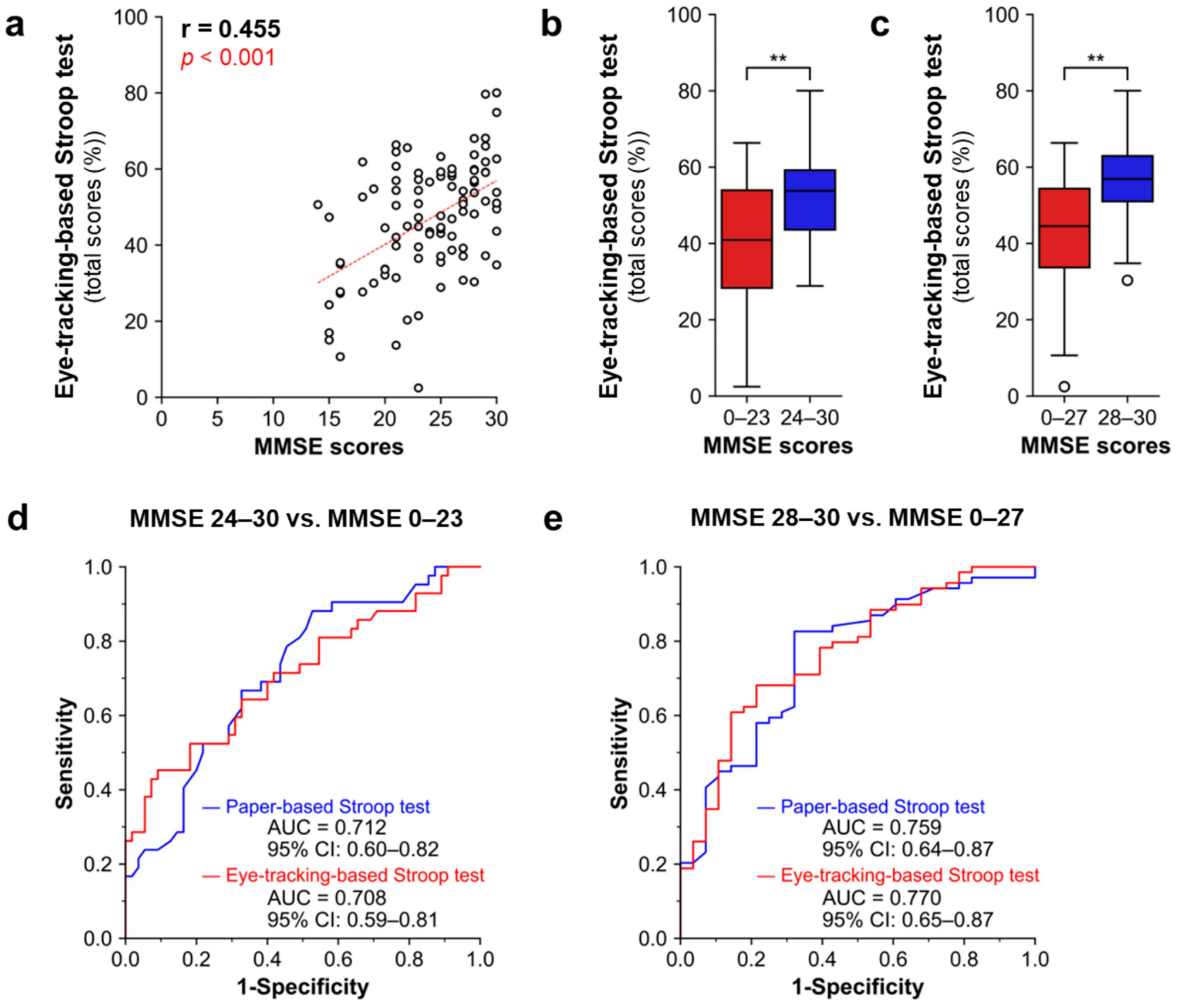
Performance of the eye-tracking-based Stroop test as a screening tool for global cognitive impairment. **(a)** Scatter plot analysis for correlations in scores between the eye-tracking-based Stroop test and MMSE. *p* < 0.001, Spearman’s rank test, *n* = 97. **(b,c)** Participants were divided into two MMSE groups, based on the traditional cutoff values for **(b)** dementia (≤23/30, *n* = 42) and **(c)** MCI (≤27/30, *n* = 69). The eye-tracking-based Stroop scores were compared between the groups. Mann–Whitney *U* test. **p < 0.01. Box plots indicate the median (horizontal line), the interquartile range (25th–75th percentiles; box), and whiskers extending to 1.5× the interquartile range. Outliers are shown as individual points. **(d,e)** ROC curve analyses of diagnostic performances on the eye tracking-based (red) and paper-based (blue) Stroop tests for **(d)** discriminating participants with suspected dementia (*n* = 42) from the non-dementia group [including MMSE-defined CN (*n* = 28) and suspected MCI (*n* = 27)], and **(e)** the participants with cognitive impairment group [including suspected MCI (*n* = 27) and dementia (*n* = 42)] from MMSE-defined CN (*n* = 28). Diagnoses of suspected MCI (MMSE score ≤ 27/30) and dementia (MMSE score ≤ 23/30) were based on the traditional cutoff values of MMSE scores. CN, cognitive normal; MCI, mild cognitive impairment; MMSE, Mini-mental state examination; ROC, receiver operating characteristic.

We next aimed to evaluate the screening performance of the eye-tracking-based Stroop test for global cognitive function and its utility for detecting MCI and dementia (Figure 6). The total scores of the eye-tracking-based Stroop test were positively correlated with MMSE scores (Figure 6a, *r* = 0.455, *p* < 0.001, Spearman’s rank test, *n* = 97), reflecting participants’ global cognitive function. Participants with MMSE scores lower than the cutoff value for dementia (≤23/30) had significantly lower scores on the eye-tracking-based Stroop test, compared to those with MMSE scores of ≥24/30 (Figure 6b). Furthermore, participants with MMSE scores lower than the cutoff value for MCI (≤27/30) had significantly lower scores in the eye-tracking-based Stroop test, compared to those with MMSE scores of ≥28/30 (Figure 6c).

We then examined the diagnostic performances of the eye-tracking-based Stroop test for detecting MMSE-defined suspected MCI and dementia (Figures 6d,e). To discriminate participants with suspected dementia from the non-demented group (MMSE-defined CN + MCI), the eye-tracking-based Stroop test achieved an AUC of 0.708 (95% CI: 0.59–0.81), which was comparable to the paper-based Stroop test (AUC = 0.712, 95% CI: 0.60–0.82) (Figure 6d). To discriminate participants with cognitive impairment (MMSE-defined suspected MCI + dementia) from MMSE-defined CN, the eye-tracking-based Stroop test achieved an AUC of 0.770 (95% CI: 0.65–0.87), which was comparable to the paper-based Stroop test (AUC = 0.759, 95% CI: 0.64–0.87) (Figure 6e).

Similar analyses were conducted for the CDR-defined CN, MCI, and dementia groups (Supplementary Figure 1). Participants in the dementia group exhibited significantly lower scores on the eye-tracking-based Stroop test compared to those in the CN and MCI groups (Supplementary Figure 1a). For discriminating participants with dementia from non-demented individuals (CN + MCI), the eye-tracking-based Stroop test achieved an AUC of 0.790 (95% CI: 0.68–0.89), while the paper-based Stroop test yielded an AUC of 0.835 (95% CI: 0.73–0.93) (Supplementary Figure 1b).

### 3.7 Potential effects of age on the eye-tracking-based Stroop test scores

We evaluated the potential effects of participant age on the test scores and correlations. While age was significantly correlated with both the eye-tracking- and paper-based Stroop test scores (Supplementary Figure 2), age-stratified analyses confirmed that significant correlations between the eye-tracking-based Stroop test and other neuropsychological assessments (the paper-based Stroop test, FAB, and TMT) were maintained even among participants in their 70s and 80s (Supplementary Figure 3).

## 4 Discussion

In this study, we developed an eye-tracking-based Stroop test and demonstrated its concurrent and construct validities by evaluating correlations of the eye-tracking-based Stroop test scores with conventional paper-based Stroop test scores (Figure 3) and neuropsychological test scores for frontal lobe function (FAB and TMT) (Figure 4). Furthermore, we demonstrated its utility as a screening tool for detecting frontal lobe dysfunction (Figure 5) and dementia (Figure 6). The test can be administered in a semi-automated manner, with minimal intervention by the examiner, which can reduce the test-related burdens (e.g., psychological stress during assessment) and inter-rater variability of the resulting scores. Notably, the test can be completed in approximately 3 min and enables the rapid assessment of frontal lobe function.

A fundamental advantage of utilizing eye-tracking metrics, specifically the percentage of viewing time on the correct option’s ROI, within the Stroop paradigm lies in the capacity to evaluate intermediate cognitive states. In conventional paper-based Stroop tests, participants provide a discrete final selection that is typically scored dichotomously (i.e., correct or incorrect). This conventional approach fails to differentiate between a correct response derived from definite cognitive resolution and one reached through uncertainty or chance, thereby lacking the capacity to evaluate intermediate cognitive states within an individual trial. In contrast, our eye-tracking-based test utilizes the raw percentage of viewing time directed at the correct option’s ROI as a continuous accuracy score. Participants are instructed to focus their gaze on the correct answer within a 7-s timeframe; rapid identification yields a relatively higher gaze percentage, whereas uncertainty or incorrect selection decreases this percentage. By capturing these intermediate states, including the degree of uncertainty during the cognitive interference process, this metric provides a more precise quantification of cognitive performance. Consequently, this continuous measurement allows for accurate performance evaluation with fewer test items, ultimately leading to shorter testing times. The robustness and reproducibility of this evaluation principle have been well-established in our previous studies (Katsuhisa et al., 2024; Oyama et al., 2019; Takami et al., 2025), and the present study demonstrates its successful application to the Stroop paradigm.

The Stroop test is one of the most widely used frontal lobe function assessments, with its neural bases and validity being extensively studied (Scarpina and Tagini, 2017). Neural regions involved in the Stroop effect, such as the dorsolateral prefrontal cortex and anterior cingulate cortex, have been reported to play key roles in conflict processing (Swick and Jovanovic, 2002). The impact of frontal lobe lesions on Stroop performance supports its validity as a frontal lobe function test (Demakis, 2004). The clinical utility of the Stroop test has also been demonstrated in diagnosing a wide range of psychiatric and neurodegenerative disorders (Johns et al., 2009; Kopp et al., 2015; Stuss et al., 2001; Valverde et al., 2009). Both age- and education level-specific reference norms have been reported for the Stroop test (Rivera et al., 2015), which enables precise interpretations of the scores. However, despite its utility and validity as a frontal lobe assessment, the test-related burdens for both examiner and examinee, including the long administration time and complicated test procedures that require the administrator’s proficiency, have been a bottleneck preventing the test from being used for routine clinical settings. The eye-tracking-based Stroop test offers the advantage of providing a shorter and more objective evaluation, thereby enhancing the clinical utility of the Stroop paradigm.

Deficits in attention and executive function are often observed as an early symptom of dementia. Objective assessments of these cognitive functions can help in detecting dementia at the early stage, such as MCI. Furthermore, Stroop test scores may reflect changes in global cognitive function and serve as a screening tool for dementia. Previous studies have reported the utility of the Stroop test in identifying both MCI and dementia (Traykov et al., 2007). In the present study, we observed that eye-tracking-based Stroop test scores were significantly correlated with total MMSE scores (Figure 6a) and achieved an AUC-ROC of 0.708 for detecting suspected dementia (Figure 6d) and of 0.770 for detecting cognitive impairment (MMSE-defined suspected MCI + dementia) (Figure 6e), which was comparable to that of the conventional paper-based Stroop test. Our eye-tracking-based Stroop test may offer a rapid, accessible, and objective way to screen for dementia.

We used a research-grade high-performance eye tracker in this study, which enabled highly accurate gaze detection. Recent advancements in digital technologies offer more accessible means of eye-tracking that work with virtual reality headsets, smart glasses, and even smartphones and tablets. The eye-tracking-based Stroop test, based on the more accessible and portable eye-tracking platform, may enhance its utility as a screening tool for frontal lobe dysfunction and dementia.

This study has potential limitations. First, the study cohort primarily consisted of older adults, ranging in age from 51 to 89 years (mean age of 76.4 years), who were enrolled in a memory clinic; therefore, age might have influenced the results of the eye-tracking-based Stroop test. Aging is broadly known to influence Stroop test performance across wider age ranges (Mathis et al., 2009). In our study, significant differences in age were observed among the MMSE-defined groups (Supplementary Table 1), and participant age was significantly correlated with both the eye-tracking- and paper-based Stroop test scores (Supplementary Figure 2). However, our age-stratified analyses confirmed that significant correlations between the eye-tracking-based Stroop test and other neuropsychological assessments were maintained even within specific age cohorts (Supplementary Figure 3), indicating that the test’s validity was not solely driven by age effects. Nevertheless, future studies collecting data from a wider, well-balanced age range are required to establish age-adjusted normative data. Additionally, because the participants were recruited from a memory clinic, our CN group does not represent typical community-dwelling controls and may include individuals with subjective cognitive decline or preclinical Alzheimer’s disease. This factor might have influenced the sensitivity and specificity of our diagnostic results. Second, we were unable to collect sufficient and reliable data regarding the participants’ educational history, which precluded us from analyzing its specific effects. Education level is a critical confounder known to have a significant impact on most neuropsychological tests, including the MMSE and the conventional paper-based Stroop test (Wang et al., 2025). Consequently, the inability to control for educational background in our analyses must be highlighted as a potential threat to the validity of the observed correlations between the eye-tracking-based Stroop test and other established measures, such as the FAB and TMT. Future studies incorporating education-adjusted analyses are essential to firmly establish the test’s validity and clinical utility. Third, we did not perform objective assessments of the participants for visual acuity or fields, although we excluded individuals with severe visual impairments who were not able to recognize the texts on the task movies. The potential effects of undetected ophthalmological disorders on the results of the eye-tracking-based Stroop test cannot be excluded. Fourth, because participants completed both the eye-tracking-based and conventional paper-based Stroop tests on the same day, we cannot rule out the potential influence of task repetition on test performance. Specifically, completing one modality first may have induced a learning effect (practice effect) or, conversely, cognitive fatigue, either of which could affect the scores of the subsequent test. As we did not systematically evaluate these potential sequence effects, this factor should be carefully considered when interpreting the concurrent validity results. Future studies should counterbalance or randomize the order of test administration to systematically verify the impact of task repetition across different modalities. Lastly, we used the MMSE to evaluate global cognitive function and classify the MMSE-defined MCI group; however, the MMSE is known to have a ceiling effect and limited sensitivity for detecting subtle cognitive decline. Future studies should incorporate more sensitive neuropsychological assessments, such as the Montreal Cognitive Assessment, to better classify MCI and to further validate the performance of the eye-tracking-based Stroop test for early dementia screening. Furthermore, the relatively small sample size (*n* = 97) may limit the generalizability of the results and the robustness of subgroup analyses, warranting future validation in larger cohorts.

Taken together, we successfully developed a rapid and objective Stroop test based on eye-tracking technology. This is a semi-automated assessment for frontal lobe function and can be completed in approximately 3 min. The test has the potential to efficiently and quantitatively assess frontal lobe function and can be used as screening tool for dementia. While we used a simple and robust eye-tracking metric (the percentage of viewing time to the correct option’s ROI) to prioritize clinical practicality, incorporating additional eye-tracking features, such as saccadic latency or fixation duration, combined with multivariate machine learning models could potentially improve the diagnostic sensitivity and specificity. Exploring these advanced analytical approaches while maintaining real-world feasibility remains an important objective for future research.

## Data Availability

The original contributions presented in the study are included in the article/Supplementary material, further inquiries can be directed to the corresponding authors.
