# Supplementary material for: An eye-tracking-based Stroop test: an efficient method for evaluating frontal lobe function
Source: Front Aging Neurosci. 2026 May 4;18:1787430. doi: 10.3389/fnagi.2026.1787430 (PMC13180897; doi:10.3389/fnagi.2026.1787430)
Supplement: Supplementary file 1 [file Data_Sheet_1.docx]

**Supplementary information**


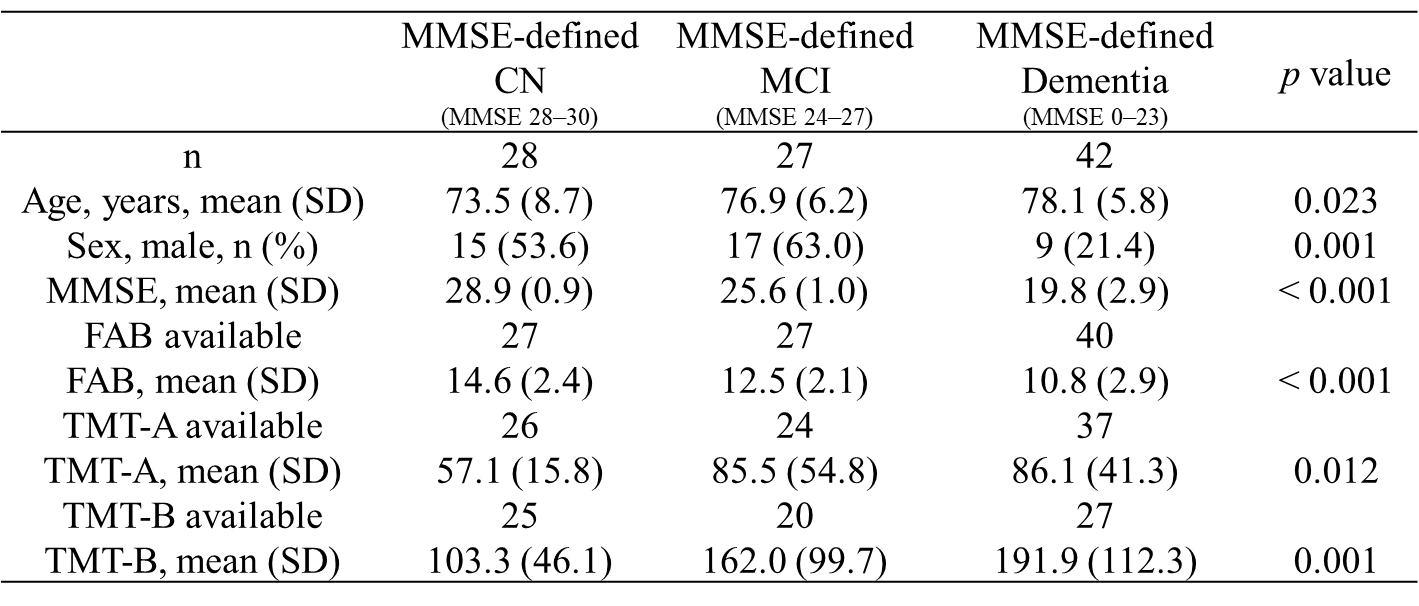


**Supplementary Table 1. Participant characteristics of the MMSE-defined CN, suspected MCI, and suspected dementia groups.**

Continuous variables (age, MMSE, FAB, TMT-A/B, CDR, and CDR-SOB) were compared using a one-way ANOVA. Sex distribution was analyzed using the Chi-square test. CDR, Clinical Dementia Rating; CN, cognitively normal; FAB, Frontal Assessment Battery; MCI, mild cognitive impairment; MMSE, Mini-Mental State Examination; TMT, Trail Making Test.


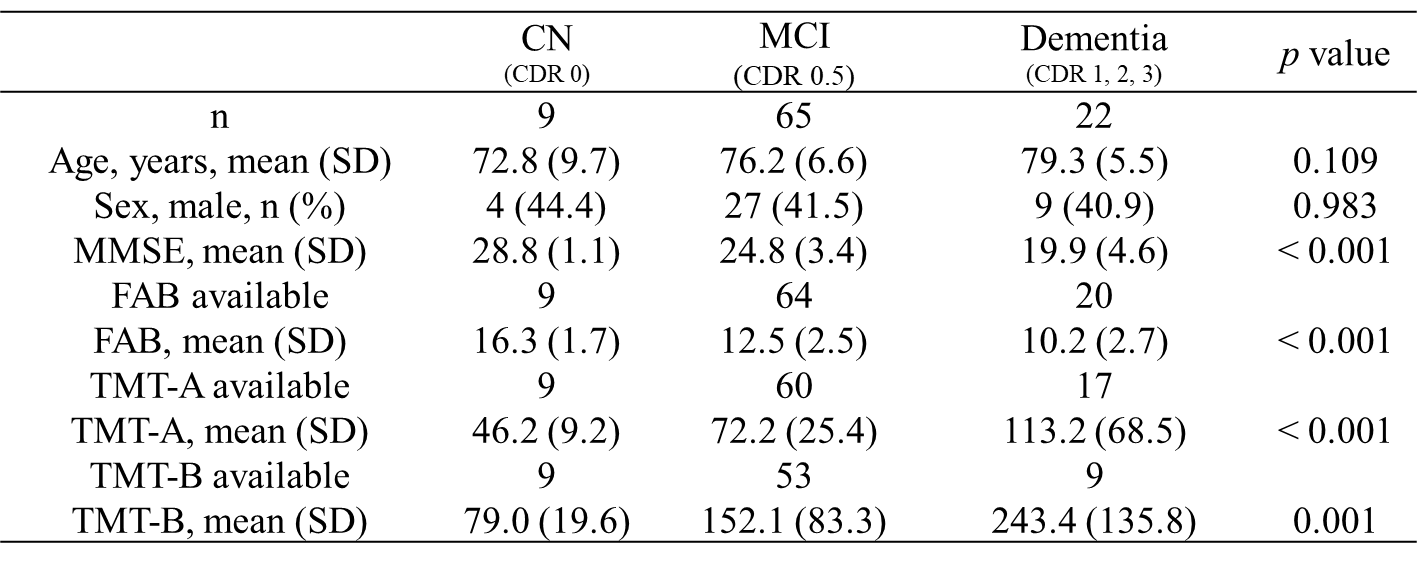


**Supplementary Table 2. Participant characteristics of the CDR-defined CN, MCI, and dementia groups.**

Continuous variables (age, MMSE, FAB, and TMT-A/B) were compared using a one-way ANOVA. Sex distribution was analyzed using the Chi-square test. CDR, Clinical Dementia Rating; CN, cognitively normal; FAB, Frontal Assessment Battery; MCI, mild cognitive impairment; MMSE, Mini-Mental State Examination; TMT, Trail Making Test.


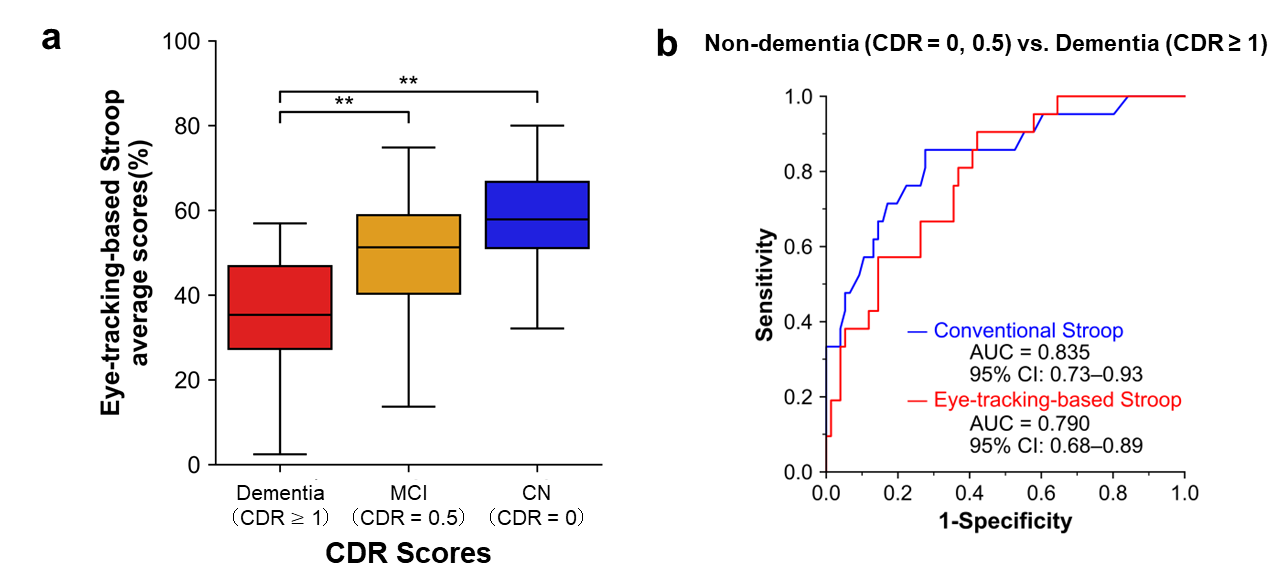


**Supplementary Figure 1. Diagnostic performance of the eye-tracking-based Stroop test for dementia**

(a) Participants were divided into three CDR-defined groups: CN (n = 9), MCI (n = 65), and dementia (n = 22). The eye-tracking-based Stroop test scores were compared among the groups. Kruskal–Wallis test followed by Dunn’s test. **p*< 0.01. Box plots indicate the median (horizontal line), the interquartile range (25th–75th percentiles; box), and whiskers extending to 1.5× the interquartile range. (b) ROC curve analyses of the diagnostic performances of the eye-tracking-based (red) and paper-based (blue) Stroop tests for discriminating participants with dementia from the non-dementia group (including CN and MCI). CDR, Clinical Dementia Rating; CN, cognitively normal; MCI, mild cognitive impairment.

**
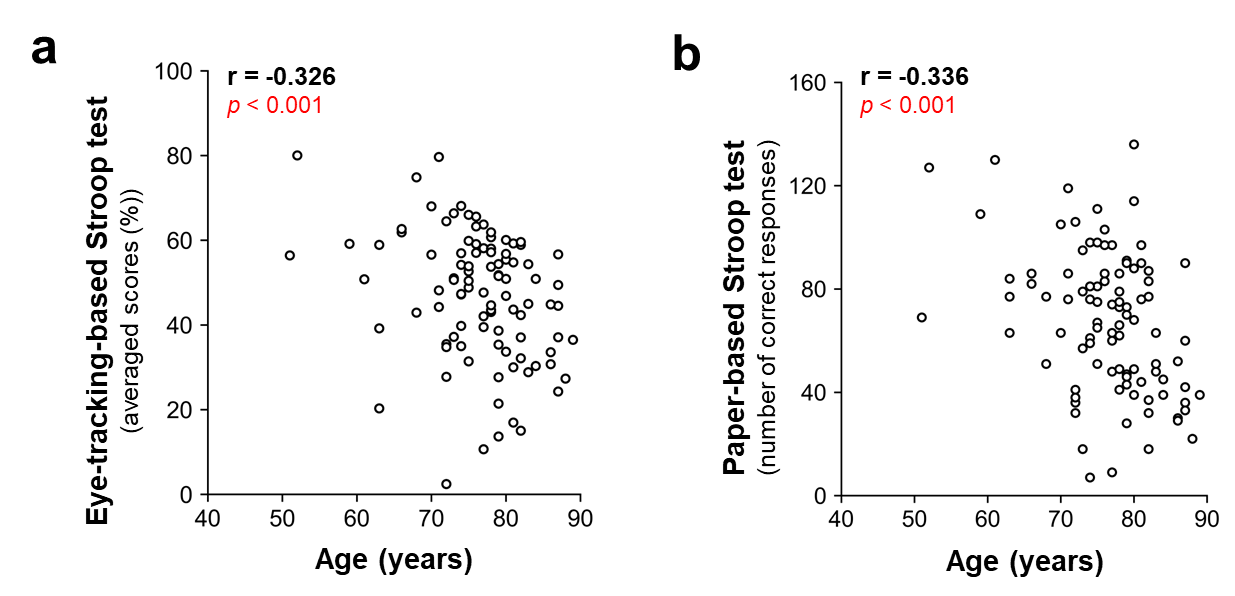
**

**Supplementary Figure 2. Correlations between age and the paper-based or eye-tracking-based Stroop test.**

(a) Scatter plot analysis for correlations in scores between the eye-tracking-based Stroop test and age. *p*< 0.001, Spearman’s rank test, n = 97. (b) Scatter plot analysis for correlations in scores between the paper-based Stroop test and age. *p*< 0.001, Spearman’s rank test, n = 97.


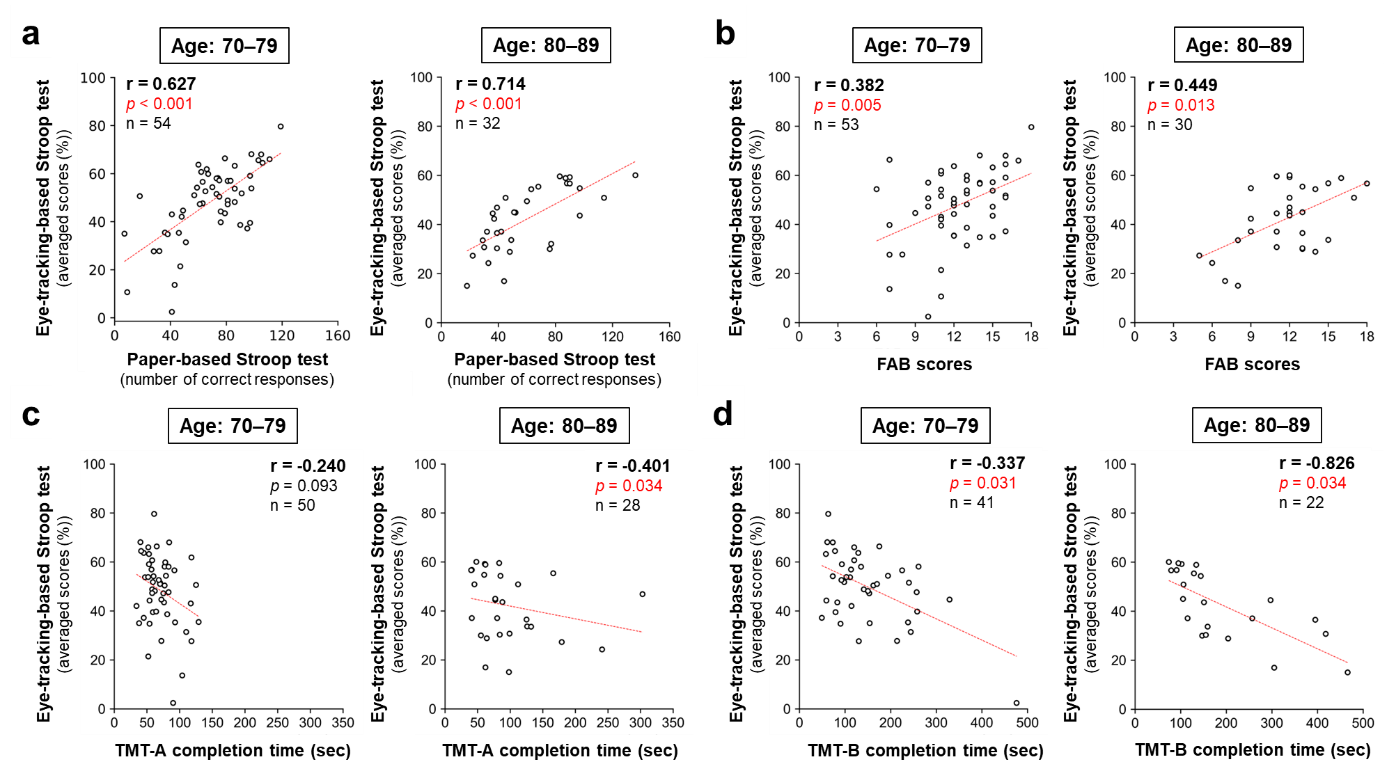


**Supplementary Figure 3. Correlations between the eye-tracking-based Stroop test and the paper-based Stroop test, FAB, TMT-A, and TMT-B in age-stratified cohorts of participants in their 70s and 80s.**

(a) Scatter plot analyses for correlations in scores between the eye-tracking- and paper-based Stroop tests in the 70s (left) and 80s (right) cohorts. Spearman’s rank test. (b) Scatter plot analyses for correlations in scores between the eye-tracking-based Stroop test and FAB in the 70s (left) and 80s (right) cohorts. *p*< 0.001, Spearman’s rank test. (c) Scatter plot analyses for correlations in scores between the eye-tracking-based Stroop test and TMT-A in the 70s (left) and 80s (right) cohorts. Spearman’s rank test. (d) Scatter plot analyses for correlations in scores between the eye-tracking-based Stroop test and TMT-B in the 70s (left) and 80s (right) cohorts. Spearman’s rank test. FAB, Frontal Assessment Battery; TMT, Trail Making Test.
